# In silico screening of alleged miRNAs associated with cell competition: an emerging cellular event in cancer

**DOI:** 10.1186/1755-8166-7-S1-P6

**Published:** 2014-01-21

**Authors:** Manish S Patel, Bhavesh V Antala, Neeta Shrivastava

**Affiliations:** 1 Department of Biotechnology, National Institute of Pharmaceutical Education and Research (NIPER)-Ahmedabad, Ahmedabad-380054, Gujarat, India; 2 Department of Pharmacognosy and Phytochemistry, B. V. Patel Pharmaceutical Education and Research Development (PERD) Centre, Ahmedabad-380054, Gujarat, India

## Background

Cell competition is identified as a crucial phenomenon for various conditions like cancer, aging and organ development. MicroRNAs (miRNAs) may play an important role in regulation of expression of genes involved in cell competition at the post-transcriptional level. In silico screening of miRNAs involved in a cell competition is an effort to identify potential miRNAs and to reduce, economize and expedite experimental work. In the present study we have identified miRNAs of Drosophila genome involved in a cell competition using in silico screening strategy.

## Material and methods

We used four steps of in silico (i) Selection of cell competition related genes of Drosophila genome through literature survey; (ii) Identification of miRNAs that target selected cell competition-related genes; (iii) Sequence conservation analysis of identified miRNAs with Human genome; (iv) Identification of genomic location of that Drosophila miRNAs and exploration of their expression profiles in tissues by use of host gene expression profile.

## Results and conclusions

In this study we have identified seven potential Drosophila miRNAs that are most probably involved in cell competition. Human homologs of these seven potential miRNAs may be very important in cell competition related diseases like cancer. Proof of this concept will be established with wet lab experimentation to reassert the role of miRNAs in cell competition.

